# Clinical Characteristics in Danish Children and Adults Diagnosed With Neuroborreliosis: A Retrospective Study From January 2016 to January 2024

**DOI:** 10.1111/ene.70250

**Published:** 2025-07-09

**Authors:** Al‐Hasan Hussein Dos, Nina Vindegaard Sørensen, Malini Vendela Sagar, Anne‐Mette Lebech, Anders Hougaard, Christian Stenør

**Affiliations:** ^1^ Department of Neurology Copenhagen University Hospital Herlev, Gentofte Denmark; ^2^ Department of Clinical Medicine, Faculty of Health and Medical Sciences University of Copenhagen Copenhagen Denmark; ^3^ Department of Infectious Diseases Copenhagen University Hospital – Rigshospitalet Copenhagen Denmark

**Keywords:** adult, *Borrelia burgdorferi* sensu lato, child, Denmark, facial paralysis, Lyme neuroborreliosis

## Abstract

**Introduction:**

Lyme neuroborreliosis (NB), is caused by tick‐borne spirochetes in the *Borrelia burgdorferi* sensu lato (*Bb*sl) genospecies complex. Although the clinical manifestations of NB in adults and children are well documented, understanding neurobiological differences between these groups can improve diagnostic accuracy and treatment approaches. This study aimed to characterize and compare the clinical presentation and cerebrospinal fluid (CSF) findings of NB in children and adults at the time of hospital admission.

**Methods:**

Retrospective analysis was performed of 3841 patients with an intrathecal *Bb*sl antibody index test performed at the Department of Microbiology at Herlev Hospital (Capital region of Denmark) between January 2016 and January 2024. Adults and children were included based on the European criteria for NB and compared for symptoms, such as peripheral facial palsy, and CSF variables, such as white blood cell (WBC) counts.

**Results:**

A total of 146 children and 267 adults were included. The annual incidence was 6.4 cases per 100 000 inhabitants. Median symptom duration before CSF analysis was 7 days for children and 21 days for adults. Facial palsy was the most common symptom in children (70%), whereas radicular pain predominated in adults (61%). CSF analysis showed significantly higher WBC counts in children vs. adults and significantly lower protein levels in children vs. adults, irrespective of symptom duration.

**Conclusion:**

There are substantial differences in the clinical presentation and CSF findings of NB between adults and children. NB incidence was much higher than previously reported in Denmark, underscoring the need for improved clinical awareness and early diagnosis.

## 1 Introduction

Lyme borreliosis (LB) is the most prevalent vector‐borne illness in the Northern Hemisphere and is caused by spirochete bacteria belonging to the *Borrelia burgdorferi* sensu lato (*Bb*sl) complex [1, 2, 3]. In Europe, there are between 650,000 and 850,000 LB cases per year, whereas there are 476,000 LB cases per year in the USA [4]. In Europe and North America, an estimated 12% of LB patients develop neurological manifestations, referred to as neuroborreliosis (NB) [3, 5]. In North America, NB is caused by the genospecies *B. burgdorferi* *sensu stricto* (*Bbss*), whereas *B. garinii* is the most common cause in Europe, with additional cases due to *B. afzelii, Bbss*, and *B. bavariensis* [3]. From 2015 to 2019, 162–200 cases of NB were diagnosed annually in Denmark, which corresponds to 2.8–3.4 cases per 100,000 inhabitants [6]. Approximately 20%–30% of these cases were among children < 18 years of age.

In Denmark and across Europe, the most common clinical manifestation of NB in adults is painful meningoradiculoneuritis with lymphocytic pleocytosis, also named Bannwarth syndrome [7, 8]. By contrast, in North America, the majority of adult NB patients present with aseptic meningitis [9]. In both European and American children, the most common clinical manifestations are peripheral facial palsy and meningitis [2, 10, 11]. These patterns are not universally consistent. In some cohorts, facial palsy was the most common manifestation in adults and meningoradiculitis the most common in children [12, 13]. Additionally, differences in clinical presentation between *Borrelia* genospecies have been observed in European NB patients [1]. Mononuclear pleocytosis and higher protein concentrations are usually found in the cerebrospinal fluid (CSF) of NB patients, with differences between adults and children [12, 14, 15, 16].

According to reports from 2010 to 2017, the capital region of Denmark is endemic for the two most common *Bb*sl genospecies in Europe, *B. afzelii* and *B. garinii* [ 17]. There is a need to better understand differences in clinical manifestations of NB between adults and children in a highly endemic area with a large human population [10, 12]. Although the 1992 study by Hansen et al. laid a foundational understanding of NB in Europe, a contemporary study is essential to capture potential changes in clinical presentation, disease progression, and outcomes due to advancements in medical practice, disease awareness and changes in the community of *Bb*sl genospecies [8].

Thus, the goal of this study was to retrospectively compare the clinical manifestations and CSF composition at admission between children and adults with definite and possible NB in a highly endemic area, highlighting key differences to improve diagnostic accuracy and reduce diagnostic delay.

## 2 Methods

### 2.1 Ethical Approval

The study was approved by the Danish health authorities according to the Danish health act paragraph 46 section 2 and paragraph 48 section 1 (J‐23033262). Ethical approval and consent from individual patients are not required for this type of study according to Danish legislation.

### 2.2 *Borrelia burgdorferi* Sensu Lato Tests

All analyses for *Bb*sl antibodies were conducted in accordance with the Danish guidelines for diagnosing LB [18].

### 2.3 Intrathecal *Borrelia burgdorferi* Sensu Lato Antibody Test

*Bb*sl antibody index (*Bb*‐AI) IgG and IgM in patient CSF were measured using a second‐generation, flagella antigen‐based capture enzyme immunoassay (IDEIA Lyme Neuroborreliosis, Oxoid, Hampshire, UK) [19]. This method determines the ratio of anti‐*Bb*sl‐specific antibodies to total antibodies of the same class in both CSF and serum. An elevated *Bb*‐AI of ≥ 0.3 was considered positive [19].

### 2.4 Serum *Borrelia burgdorferi* Sensu Lato Antibodies

*Bb*sl antibodies in patient serum were assessed using LIAISON *Borrelia* Immunoglobulin G (IgG)/*Borrelia* Immunoglobulin M (IgM) Quant (DiaSorin, Italy). Since May 2017, only *Bb*sl IgG values were measured.

### 2.5 Inclusion Process

Data was retrospectively gathered from the electronic health records (EHR) of 3841 patients who had a *Bb*‐AI test performed at the Department of Microbiology at Herlev and Gentofte Hospital (HGH) between January 2016 and January 2024 in the Capital Region of Denmark, which has approximately 800,000 inhabitants. Patient data on the admission date, tick exposure, clinical symptoms, CSF parameters, and *Bb*sl serology were collected and stored in a REDCap database (v.14.1.2) [20]. Adulthood was defined as age at admission ≥ 18 years. Definite NB was defined as fulfillment of all the following criteria: (1) neurological symptoms consistent with NB, (2) white blood cell count (WBC) > 5 WBCs/μL in CSF, and (3) positive Bb‐AI test [21]. Neurological symptoms consistent with NB included peripheral nervous system (PNS) manifestations (e.g., painful meningoradiculitis, lymphocytic meningitis, plexus neuritis, mononeuritis multiplex, mononeuropathy, and polyneuropathy) and central nervous system (CNS) manifestations (e.g., myelitis, encephalitis, cerebral vasculitis, and encephalomyelitis) [7, 21]. If only two out of three criteria were met, the patient was classified as having possible NB. In cases with CSF pleocytosis and symptoms suggestive of NB but with a negative *Bb*‐AI, a positive serum *Bb*sl antibody test was required to meet the criteria for possible NB, provided the symptoms persisted for more than 6 weeks [21].

Patients who (1) fulfilled criteria for definite or possible NB, (2) had a lumbar puncture (LBP) at admission, and (3) were admitted to either HGH or North Zealand Hospital (both serviced by HGHs Department of Microbiology) were included in the study. Patients who (1) had another more probable diagnosis or (2) limited information on neurological symptoms in the HER were excluded.

### 2.6 Statistics

All statistical analyses were performed with R (version 2024.4.0.735) [22]. A *p*‐value of < 0.05 was considered statistically significant. Categorical variables were summarized as counts with percentages, compared with odds ratios with 95% confidence intervals (CI), and tested with Chi‐squared (*χ* ^2^) tests. Means and standard deviations (SD) were calculated for continuous variables with a normal distribution. For skewed data, we calculated the median and range. Continuous variables with a normal distribution were compared using an independent two‐sample *t*‐test, whereas the Mann–Whitney *U* test was applied for variables with a skewed distribution. Analysis of covariance (ANCOVA) was used to compare the continuous CSF variables between adults and children while controlling for the duration of symptoms (a potential confounding covariate), following the approach of Tveitnes et al. [23].

The radar chart showing the distribution of symptoms and the histograms showing the seasonal patterns in the months of onset of symptoms, hospital admission, and treatment were made with Microsoft Excel (v16.0). Graphical additions to the histograms were made with Biorender (https://www.biorender.com).

## 3 Results

Of the 3841 patients with sufficient electronic data and who had a *Bb*‐AI test between January 2016 and January 2024, 477 patients fulfilled the criteria for possible or definite NB. Two cases, who otherwise fulfilled criteria for definite NB, were excluded because herpes simplex virus 2 sacral meningoradiculitis was a more probable diagnosis based on clinical signs and a positive intrathecal herpes simplex test. Sixty‐four cases, who otherwise fulfilled criteria for possible NB, were excluded because of a more probable alternative diagnosis, including one with subdural empyema, one with hypoglossal nerve palsy, one with peroneal nerve palsy, two with herpes simplex virus 1 encephalitis, three with unclassified encephalitis, seven patients with demyelinating diseases, 11 with tick‐borne encephalitis, 18 with other aseptic meningitis, 22 with Ramsay Hunt syndrome. Thus, a total of 413 cases were included, of which 267 were adults: 177 classified as definite NB and 90 as possible NB. The remaining 146 cases were children, with 91 classified as definite NB and 55 as possible NB. The annual incidence was 6.4 cases of NB per 100,000 individuals in the Capital Region of Denmark between January 2016 and January 2024.

### 3.1 Baseline Characteristics

**FIGURE 1:**
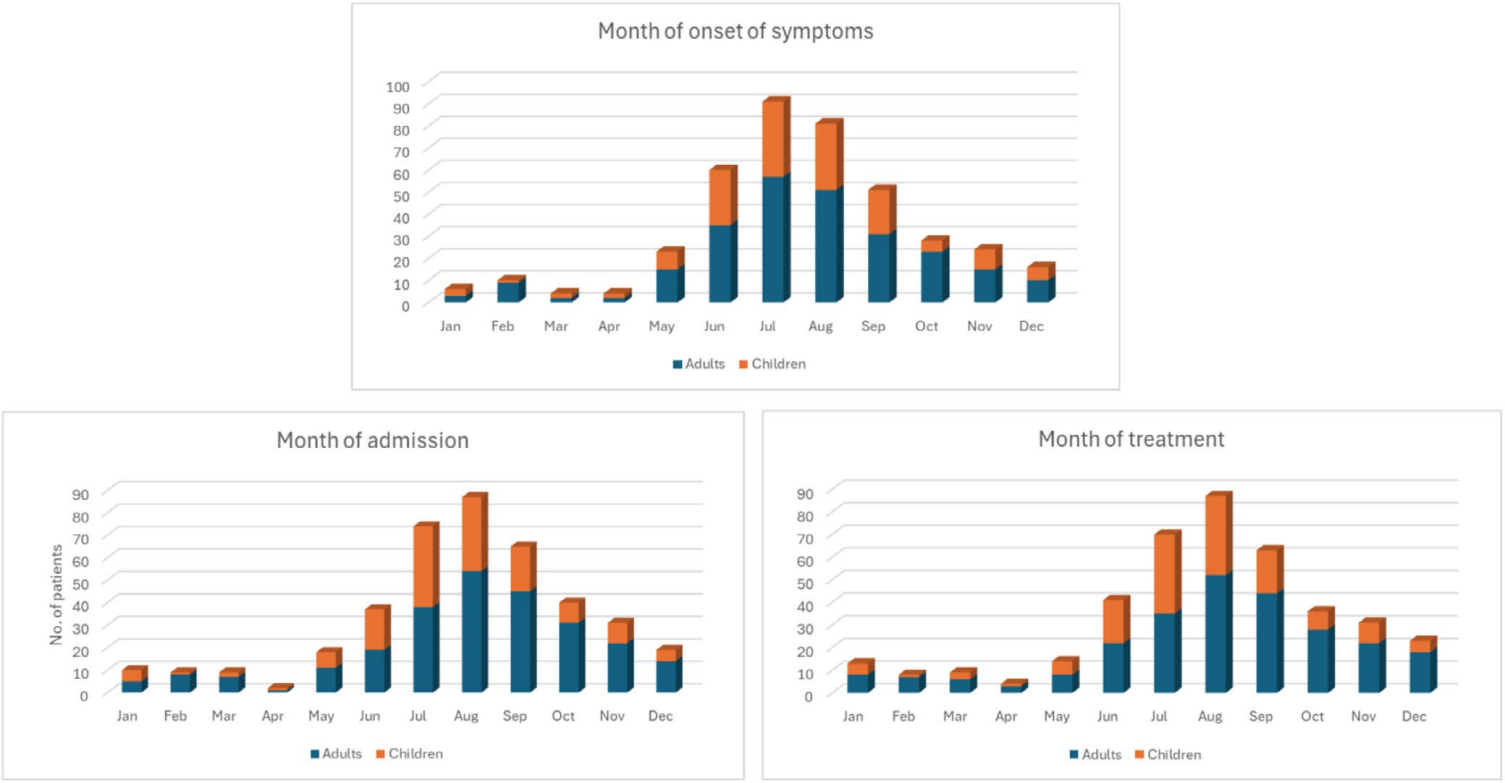
Seasonal variation of adults and children with neuroborreliosis from the Capital region of Denmark, January 2016–January 2024. Data was available on 398/413 patients for the onset of symptoms, 401/413 for the admission date, and 399/413 for the month of treatment. Time of treatment was counted from when patients received relevant antibiotic treatment against neuroborreliosis.

Demographics of the included NB patients are presented in Table 1. All children were admitted to a pediatric emergency department (ED). Of the 267 adult NB patients, 58% (156/267) were admitted to a neurological ED, whereas 37% (98/267) were admitted to the Department of Internal Medicine. Six adult patients were initially admitted to Gastroenterology units because of abdominal pain. These six patients received an abdominal CT scan, and three patients received an endoscopy without significant findings. One patient was initially admitted to a cardiac ward, because of chest pain. The remaining six adult patients were admitted to other departments. Median symptom duration before LBP was 7 days for children (range: 0–135 days) and 21 days for adults (range: 0–1000 days) and this difference was significant (*p* < 0.001). In children presenting with headache, the median symptom duration was 14 days before CSF examination (range: 0–110 days). Figure 1 shows the seasonal distribution of onset of symptoms, hospital admission and antibiotic treatment initiation of our cohort. For both adults and children, symptom onset peaked in the summer months (June–August).

**TABLE 1:** Baseline and clinical characteristics of 413 patients with neuroborreliosis from the Capital region of Denmark, January 2016–January 2024.

	Total	Children	Adults ≥ 18 years of age	Odds ratio	*p*
Number included, *N* (%)	413 (100%)	146 (35%)	267 (65%)		
Definite NB^a^	268 (65%)	91 (62%)	177 (66%)		0.485
Possible NB^b^	145 (35%)	55 (38%)	90 (34%)		*—*
Median symptom duration before LBP (range), days	15 (0–1000)	7 (0–135)	21 (0–1000)		< 0.001
*Demographics, N (%)*					
Age (years), median (range)	51 (2–86)	7 (2–17)	63 (18–86)		< 0.001
Female (%)	210 (51%)	73 (50%)	116 (43%)		0.240
*Borrelia exposure, N (%)*					
Reported tick bite	168 (41%)	60 (41%)	108 (40%)		0.981
Reported forest/green area exposure	222 (54%)	77 (53%)	145 (54%)		0.839
Reported rash	128 (31%)	38 (26%)	90 (34%)		0.133
*Neurological symptoms*					
Peripheral facial palsy	205 (49%)	103 (70%)	102 (38%)	3.87 (2.51, 5.98)	< 0.001
Peripheral facial palsy without any pain^c^	53 (26%)	45 (44%)	8 (8%)	9.12 (2.77, 11.22)	< 0.001
Peripheral facial palsy with radicular pain^c^	72 (35%)	9 (9%)	63 (62%)	0.06 (0.03, 0.013)	< 0.001
Peripheral facial palsy and headache	73 (18%)	36 (25%)	37 (14%)	2.03 (1.22, 3.39)	0.009
CN VI palsy	10 (1%)	6 (4%)	4 (1%)	2.82 (0.78, 10.15)	0.188
Combined cranial nerve palsy^d^	3 (1%)	0 (0%)	3 (1%)	N/A	0.496
Meningitis (headache + fever)	61 (15%)	40 (27%)	21 (8%)	4.40 (2.48, 7.83)	< 0.001
Dysesthesia	150 (36%)	12 (8%)	138 (52%)	0.08 (0.04, 0.16)	< 0.001
Paresis of extremities	60 (14%)	3 (2%)	57 (21%)	0.008 (0.02, 0.25)	< 0.001
*Pain*					
Any pain excluding headache	295 (71%)	67 (46%)	228 (85%)	0.15 (0.09, 0.23)	< 0.001
Headache	168 (41%)	74 (51%)	94 (35%)	1.89 (1.25, 2.85)	0.003
Radicular pain^e^	188 (45%)	25 (17%)	163 (61%)	0.13 (0.08, 0.22)	< 0.001
Lower back pain	103 (25%)	11 (7%)	92 (34%)	0.12 (0.06, 0.23)	< 0.001
Abdominal pain	56 (13%)	24 (16%)	32 (12%)	1.44 (0.81, 2.56)	0.265
*General symptoms*					
Fever^f^	100 (24%)	60 (41%)	40 (15%)	3.96 (2.47, 6.34)	< 0.001
Nausea or vomiting	79 (19%)	29 (20%)	50 (19%)	3.96 (2.47, 6.34)	0.880
Fatigue	103 (25%)	53 (36%)	50 (19%)	2.47 (1.57, 3.90)	< 0.001
Weight loss	54 (13%)	16 (11%)	38 (14%)	0.74 (0.40, 1.38)	0.429

Abbreviations: CN VI, abducens nerve; LBP, lumbar puncture; *N*, number; N/A, not applicable; NB, neuroborreliosis.

^a^ Definite NB: 3/3 criteria – Neurological symptoms suggestive of NB, CSF pleocytosis (> 5 leukocytes/μL), intrathecal antibody synthesis.

^b^ Possible NB 2/3 of the criteria‐ after 6 weeks, if intrathecal synthesis is lacking, then a positive serum IgG is required. A total of 137 possible NB patients had pleocytosis and symptoms suggestive of NB without a more probable cause (and no intrathecal *Bb*sl antibody synthesis), whereas eight patients had a positive intrathecal borrelia test and symptoms suggestive of NB without a more probable cause (without pleocytosis).

^c^ Percentage out of the patients with peripheral facial palsy.

^d^ Two patients suffered from combined facial and abducens palsy, and one patient suffered from combined abducens nerve and oculomotor palsy.

^e^ Radicular pain is projected or moves down the arm or leg or neck, and lower back to other parts of the body.

^f^ Reported by patients before admission. Includes both reported subjective feelings of fever (feeling warm or cold and shivering) and measured temperatures of > 38°C with a thermometer at home.

### 3.2 Comparison of Symptoms Between Children and Adults

**FIGURE 2:**
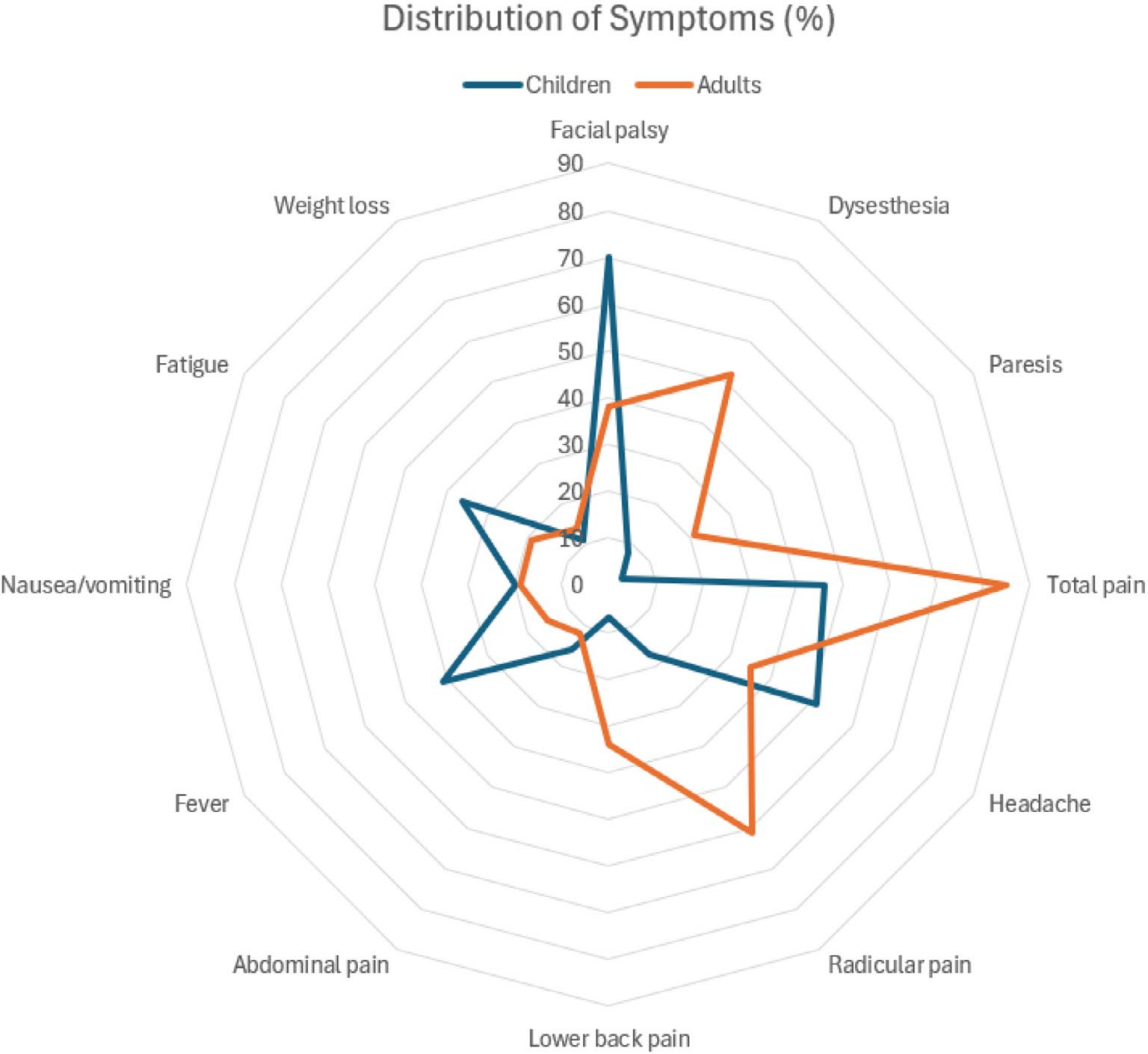
Radar chart showing relative percentages of symptoms among 413 adults and children with neuroborreliosis from the Capital region of Denmark, January 2016–January 2024.

Comparison of symptoms is presented in Table 1 and Figure 2. In children, the most common symptom was peripheral facial palsy (70% (103/146) of children compared to 38% (102/267) of adults, *p* < 0.001), followed by headache (51% (74/146) of children compared to 35% (94/267) of adults, *p* = 0.003), and any type of pain (46% (67/146) of children compared to 85% (228/267) of adults, *p* < 0.001). Children had a significantly higher percentage of non‐specific symptoms, including fever (41%, 60/146) and fatigue (36%, 53/146) compared to 15% (40/267) and 19% (50/267) of adults, respectively (*p* < 0.001). Children reported meningitis symptoms, including headache and fever (27%, 40/146), more often than adults (8%, 21/267) (*p* < 0.001). Children also had a higher percentage of headache combined with facial palsy (25%, 36/267) compared to adults (14%, 37/267) (*p* = 0.009). Of patients with facial palsy, 44% (45/103) of children reported no pain compared to 8% (8/102) of adults (*p* < 0.001).

Among adults, 61% (163/267) reported radicular pain compared to 17% (25/146) of children (*p* < 0.001). Dysesthesia was reported in 52% (138/226) of adults compared to 8% (12/146) of children (*p* < 0.001). Paresis was found in 21% (57/267) of adults, thus, much higher than the 2% (3/146) of children (*p* < 0.001). Of patients with facial palsy, 62% (63/102) of adults reported radicular pain compared to 9% (9/103) of children (*p* < 0.001).

### 3.3 Cerebrospinal Fluid Findings

CSF parameters are presented in Table 2 and comparisons of CSF laboratory results, adjusted for symptom duration, in Table 3. In children, the median WBC count in CSF was 122/μL (range: 1–1330/μL) compared to 84/μL (range: 0–1350/μL) in adults (*p =* 0.004). Mean CSF protein level was significantly lower in children compared to adults (*p* < 0.001). Adults had a higher median CSF albumin and CSF/blood albumin ratio (Q‐Alb) compared with children (*p* = 0.001 and *p* < 0.001, respectively). The mean IgG index was significantly higher in adults (*p* = 0.006).

**TABLE 2:** Cerebrospinal fluid findings in patients with neuroborreliosis from the Capital region of Denmark, January 2016–January 2024.

	Total (*N* = 413)	Children (*N* = 146)	Adults (*N* = 267)	*p*	Number of observations^a^
Median WBC count (range) (ref: 0–4)/μL [24]	93 (0–1350)	122 (1–1330)	84 (0–1350)	0.004	411/413
Median WBC mononuclear cell percentage (range)	98% (54%–100%)	98% (54%–100%)	99% (55%–100%)		372/413
Mean protein level ± SD (ref: 0.2–0.6)^b^ g/L	1.09 ± 0.80	0.72 ± 0.56	1.28 ± 0.84	< 0.001	400/413
Mean CSF/blood glucose ± SD (ref: 0.48–0.87)^d^	0.55 ± 0.13	0.59 ± 0.11	0.52 ± 0.13	< 0.001	321/413
Median albumin (range) (ref: 110–350) mg/L	511 (77–2850)	218 (77–1290)	668 (115–2850)	0.001	73/413
Median Q‐Alb (ref: < 6.3)^c^	13.2 (1.8–72.0)	5.5 (1.8–30.0)	16.9 (2.7–72)	< 0.001	74/413
Mean lactate ± SD (ref: 1.3–2.5)^d^ mmol/L	2.3 ± 0.73	1.9 ± 0.4	2.4 ± 0.7	< 0.001	135/413
Median IgG (range) (ref: 10–40) mg/L [25]	98 (7–700)	34 (7–300)	122 (15–700)	0.001	79/413
Mean IgG index ± SD (ref: < 0.63)^e^	0.81 ± 0.50	0.60 ± 0.20	0.86 ± 0.55	0.006	69/413

*Note:* Normally distributed continuous variables compared with independent two sample *t*‐test and continuous variables, with skewed data, compared with Mann–Whitney *U* test.

Abbreviations: CSF, cerebrospinal fluid; IgG, immunoglobulin G; LBP, lumbar puncture; N, number; NB, neuroborreliosis; Q‐Alb, albumin index; ref., reference range; SD, standard deviation; WBC, white blood cell.

^a^ Number of patients with available data.

^b^ 50–55‐year‐old category [26].

^c^ 18–44‐year‐old category [27].

^d^ 50–60‐year‐old category [28].

^e^ 18–88‐year‐old category [27].

**TABLE 3:** Analysis of covariance (ANCOVA)—symptom duration as a confounder for CSF‐WBC count, protein levels, albumin, glucose, lactate, and IgG in the CSF.

	Estimate	Std. Error	*t*	*p* ^a^
*WBC*				
Intercept	143.20	12.55	11.40	< 0.001
Children	41.13	19.65	2.09	0.037
Symptom duration	−0.16	0.11	−1.41	0.15
*Protein level*				
Intercept	1.27	0.05	23.78	< 0.001
Children	−0.54	0.08	−6.45	< 0.001
Symptom duration	0.0004	0.0004	0.82	0.41
*CSF/blood glucose*				
Intercept	0.52	0.01	54.77	< 0.001
Children^b^	0.06	0.01	4.32	< 0.001
Symptom duration	−0.0001	0.00008	−1.52	0.12
*Q‐Alb*				
Intercept	21.08	2.12	9.90	< 0.001
Children	−10.95	4.03	−2.71	0.008
Symptom duration	−0.01	0.014	−0.79	0.42
*IgG index*				
Intercept	0.92	0.08	11.26	< 0.001
Children	−0.29	0.15	−1.92	0.05
Symptom duration	−0.0006	0.0005	−1.28	0.20
*CSF lactate*				
Intercept	2.47	0.09	28.67	< 0.001
Children	−0.52	0.14	−3.64	< 0.001
Symptom duration	−0.001	0.001	−1.18	0.23

Abbreviations: CSF, cerebrospinal fluid; IgG, immunoglobulin G; Q‐alb, albumin index; Std. Error, standard error; WBC, white blood cell.

^a^ *p*‐value from analysis of covariance (ANCOVA).

^b^ Children were contrasted against adults where adults were the reference group in the analysis. For example, the protein level for the children – adult contrast is −0.54, which indicates that children have lower protein levels than adults.

Levels of WBC, CSF protein, lactate, CSF/blood glucose ratio, and Q‐Alb differed significantly between children and adults after adjusting for symptom duration as a confounder (all *p‐*values ≤ 0.006). However, no significant difference was observed in the IgG index (*p* = 0.05) (Table 2).

With ANCOVA, no significant relationship was found between WBC count and symptom duration in children and adults (*p* = 0.186 and *p* = 0.077, respectively).

## 4 Discussion

To our knowledge, this study is the largest of its kind to compare symptoms and CSF findings between children and adults with NB. Most children presented with facial palsy or symptoms of lymphocytic meningitis, whereas the most common presentation among adults was radicular pain.

The median symptom duration before LBP was three times longer for adults compared to children. In a previous study, patients with peripheral facial palsy were hospitalized earlier than patients with polyradiculitis [29]. Similarly, meningitis symptoms might prompt earlier hospitalization compared to other clinical presentations. Thus, the difference in time from symptom onset until diagnostic testing may be explained by the differences in symptom presentation between the groups. This hypothesis is supported by our seasonal graphs for adult patients showing a slight skew of admission and treatment toward the autumn and winter months (October–February) despite symptom onset peaking during the summer months (June–August). A treatment delay has previously been observed between November and April for both adults and children [14, 15]. In the study by Hansen et al., despite similar clinical manifestations in adults and children to our study, the median disease duration before diagnosis was 18 days in children compared to 7 days in our study [8]. This difference might indicate improvements in NB awareness over the last 25 years, leading to more efficient diagnosis.

### 4.1 Neuroborreliosis in Children

Facial palsy and headache were the two most common symptoms in children consistent with previous findings [2, 10]. However, a French study reported that the majority of children (62%, 16/26) with NB presented with meningoradiculitis [13]. In the French study, NB patients were defined by the criteria of the French Infectious Disease Society (SPILF) (in which NB can be diagnosed on clinical presentation and a positive serum *Bb*sl antibody test alone) rather than the criteria of the European Federation of the Neurological Society (EFNS) that we based our study on [21]. The large variation in prevalence of radicular pain between the different studies might be explained by the term “radicular pain” tself. A review showed that there exists considerable variation in the prevalence of radicular/sciatic pain between studies, partly because of inconsistent definitions of this term [30]. Most comparable studies do not present their definition of radicular pain [10, 12, 14, 15]. The difference in pain distribution between adults and children may be partly explained by the communication challenges of younger children. As children grow older, their ability to reliably express pain improves [31]. Additionally, their understanding of complex pain concepts develops with age [32].

A significantly higher percentage of children reported symptoms suggestive of meningitis compared to adults, corroborating a Polish study from 2020 [12].

The significant difference in the proportion of facial palsy patients without pain in our cohort (44% of children vs. 8% of adults) emphasizes that the absence of pain in children cannot reliably be used to differentiate facial palsy due to NB from other causes, such as Bell's palsy, or acoustic neuromas [33].

Avery's “rule of 7's” is a proven algorithm for differentiating NB from other aseptic meningitis in children [34, 35]. Children with < 7 days of headache, < 70% mononuclear CSF WBC and no facial palsy are at a lower risk of NB. Our cohort fits the description of high‐risk patients (14 days of headache before LBP, 98% mononuclear cells in the CSF and a high percentage of facial palsy). However, only 25% (36/146) of children in our cohort had a combination of headache and peripheral facial palsy. This combination was as high as 60% in other cohorts [36]. We did not include a control group of non‐NB patients, so we cannot assess the accuracy of the algorithm in our cohort.

### 4.2 Neuroborreliosis in Adults

Our results for adults were also consistent with earlier studies, including a nationwide Danish prospective cohort study from 2020 [14], which included 194 adult patients from 2015 to 2017. In that study, 70% of the patients suffered from radicular pain compared to 61% of our adult patients. Similarly, 35% of our adult cohort complained of headache compared to 38% of their cohort. A similar percentage of adult patients suffered from extremity paresis in their study versus our study (17% vs. 21%). In general, our study highlights the significant role of pain in diagnosing NB in adults compared to children, as evidenced by 62% of adult facial palsy patients suffering from radicular pain compared to only 9% of children.

In comparison to a retrospective Danish cohort study of 431 patients with NB, including both children and adults, our study found higher prevalence of abdominal pain (13% vs. 0.5%), weight loss (13% vs. 5.3%), and fatigue (25% vs. 15%) [15]. Most likely, these differences between children and adults reflect that data on patient symptoms depend on the patient's ability to communicate their symptoms. A prospective study would allow for a more systematic and reproducible approach to extracting information regarding symptoms. The true number of patients suffering from fatigue, abdominal pain, or weight loss could therefore be underrepresented in our study and the study from 2020 [15]. However, since we made a thorough review of each patient's medical records, we might have found symptoms not thought of in a prospective study, such as abdominal pain.

### 4.3 Differences Between Neuroborreliosis in Children and Adults

#### 4.3.1 Pathophysiology Behind the Difference in Symptom Presentation Between Adults and Children

The mechanisms by which *Bb*sl causes symptoms, such as paresis and pain include direct cytotoxicity, neurotoxic mediators, and/or autoimmune reactions, leading to inflamed and swollen nerves, as demonstrated by biopsies, ultrasound, and magnetic resonance imaging (MRI) [3, 37, 38, 39]. Although most cases of inflamed cranial nerves do not exhibit corresponding clinical symptoms, inflammation within the distal internal auditory canal is a notable exception, as it frequently leads to facial nerve palsy due to nerve entrapment in this confined space [3, 39]. Narrowing of the facial canal has been suggested as a risk factor for idiopathic facial palsy, and differences in the facial nerve to facial canal ratio and meatal width between adults and children may help explain why facial palsy is more common in children with NB [40, 41].

Additionally, animal studies have shown age‐related differences in glial activation and inflammatory responses [42]. In adult mice and rats, neuropathic pain is linked to glial activation and pro‐inflammatory responses, which sensitize neurons in the spinal cord [42]. In contrast, juvenile rodents show a weaker glial response and increased anti‐inflammatory cytokines following nerve injury. In summary, this age‐related variation in neurobiology in rodent models may explain the observed differences in neuropathic pain and radicular pain between human adults and children [42].

Moreover, the decrease in intervertebral foramen height and the proximity of nerve roots to the foramen with age could increase the likelihood of inflamed nerve root impingement in adults, further contributing to the higher prevalence of radicular symptoms in adult NB patients [43]. These hypotheses require further study to clarify the mechanisms behind the observed differences.

#### 4.3.2 WBC and Protein Levels in CSF

In our study, CSF protein levels were lower and WBC counts higher in children compared to adults, similar to a previous study [12]. When adjusting for symptom duration, differences in CSF WBC counts and protein levels were found between children and adults. Reports have shown that CSF protein concentration (specifically albumin) is positively correlated with body mass index (BMI) and age [44]. Due to lack of reliable data on patient weight and height in the medical records used in our study, we were not able to adjust for BMI in our analyses.

In our study, we found no significant relationship between CSF WBC count and symptom duration in our NB patients (children and adults). In contrast, the 2009 study by Tveitnes et al. found a positive relationship between the CSF WBC count and symptom duration in children with NB [23]. One explanation for the difference between these two studies is that Tveitnes et al. [23] focused on the first 60 days of symptom duration, whereas in our study, symptom duration for children and adults ranged up to 135 and 1000 days, respectively. This difference suggests that the positive relationship between CSF WBC count and symptom duration may exist over acute infection (i.e., < 60 days), but may disappear over chronic infection.

#### 4.3.3 CSF Albumin and IgG

The CSF composition depends on the different manifestations of NB. Manifestations of NB, such as meningitis and cranial nerve palsy, have been shown to have significantly lower Q‐Alb compared to vasculitis and encephalomyelitis [45]. In our study, adults had significantly higher albumin levels than children (*p* = 0.001), likely reflecting the different NB manifestations, as children more often presented with meningitis and cranial nerve palsies. A key finding is that Q‐Alb remained significantly higher in adults, even after adjusting for symptom duration, whereas there was no such age group‐specific difference for the IgG index.

Elevated Q‐Alb and IgG indices both indicate blood–brain barrier (BBB) disruption and increased permeability [46]. The Q‐Alb, in particular, is associated with inflammatory markers, such as interleukin‐6, and matrix metalloproteinases, which contribute to BBB disruption [46]. However, a study on cytokine profiles in NB patients did not find age‐dependent changes in CSF cytokines, suggesting that the increased BBB permeability in adults may not be driven solely by cytokine differences [47].

Nevertheless, the higher Q‐Alb in adults aligns with studies in rodents and humans that show increased BBB permeability with aging, especially during CNS inflammation [48, 49]. This is corroborated by our findings of higher Q‐Alb and IgG indices than their respective reference values in adults but not in children [50]. The significance of Q‐Alb is further highlighted by a 2014 cohort study, which found that Q‐Alb was markedly elevated between inflammatory and non‐inflammatory CNS conditions, whereas the IgG index did not differ significantly between these two conditions [24].

### 4.4 Strengths and Limitations

The major strength of this study is the number of participants, combined with a high degree of detail in the reporting of clinical manifestations and CSF findings. We also had strict criteria for the diagnosis and inclusion of NB patients, and the same observer reviewed all the patient charts, which ensured a uniform inclusion process. Our study is based in an area endemic for *B. afzelii* and *B. garinii* , the two dominant *Bb*sl genospecies that cause LB in Europe (reflected by the higher incidence compared to the reported national incidence in Denmark) and is therefore highly relevant to the people of Denmark and the rest of Europe [17]. However, like most clinical studies on NB, a limitation of our work is the absence of data on the specific *Bbsl* genospecies that infected our patients. Additionally, the retrospective nature of the study poses constraints, including incomplete data on parameters, such as albumin, Q‐Alb, IgG, and IgG index.

## 5 Conclusions

In conclusion, in patients with NB, peripheral facial palsy and lymphocytic meningitis were the most common symptoms in children, whereas radicular pain was the most common symptom in adults. Despite the retrospective nature of the study, the large patient population from a highly endemic area provides robust insights into the age‐related differences in clinical manifestations of NB in Europe, thereby helping clinicians to better recognize the most common and lesser‐known symptoms of NB and potentially improving the timeliness and accuracy of treatment decisions.

## Author Contributions

**Al‐Hasan Hussein Dos:** software, formal analysis, project administration, methodology, writing – review and editing, conceptualization, investigation, writing – original draft, data curation, visualization, resources, validation. **Nina Vindegaard Sørensen:** software, formal analysis, writing – review and editing, conceptualization, validation, methodology, project administration. **Malini Vendela Sagar:** software, formal analysis, methodology, conceptualization, visualization. **Anne‐Mette Lebech:** supervision, writing – review and editing, validation, visualization. **Anders Hougaard:** writing – review and editing, supervision, conceptualization, visualization, validation. **Christian Stenør:** funding acquisition, conceptualization, methodology, supervision, visualization, project administration, resources, validation, data curation, writing – review and editing.

## Conflicts of Interest

Anne‐Mette Lebech reports speakers' honorarium/travel grants/advisory board activity and unrestricted grant from Gilead, speakers honorarium/travel grants from GSK, speaker's honorarium/advisory board activity from Pfizer outside this work. The other authors have no conflicts of interest to declare.

## Data Availability

The data that support the findings of this study are available on request from the corresponding author. The data are not publicly available due to privacy or ethical restrictions.
