# Correction to “Clinical Characteristics in Danish Children and Adults Diagnosed With Neuroborreliosis: A Retrospective Study From January 2016 to January 2024”

**DOI:** 10.1111/ene.70346

**Published:** 2025-08-29

Dos, Al‐Hasan Hussein, et al. “Clinical Characteristics in Danish Children and Adults Diagnosed With Neuroborreliosis: A Retrospective Study From January 2016 to January 2024.” *European Journal of Neurology*, vol. 32, no. 7, 2025, pp. e70250, https://doi.org/10.1111/ene.70250.

In this article, the following errors were identified in Table 2:

An incorrect reference was used for the WBC reference range.

The correct reference should have been: Rahimi, Jasmin, and Adelheid Woehrer. “Overview of Cerebrospinal Fluid Cytology.” *Handbook of Clinical Neurology*, vol. 145, Elsevier Health Sciences, 2018, pp. 563–71, https://doi.org/10.1016/B978‐0‐12‐802395‐2.00035‐3.

An incorrect reference was used for Q‐albumin and IgG index reference ranges.

The correct reference should have been: Blennow, K., et al. “Protein Analysis in Cerebrospinal Fluid. II: Reference Values Derived from Healthy Individuals 18–88 Years of Age.” *European Neurology*, vol. 33, no. 2, 1993, pp. 129–33, https://doi.org/10.1159/000116919.

The correct upper reference limit for Q‐albumin should have been < 6.8.

The reference range for protein should have been 0.2–0.5 g/L.

In the footnotes, the age category for protein should have been 5–60 years.

We apologize for these errors.

